# The Ophthalmoscope

**Published:** 1892-09

**Authors:** 


*The Ophthalmoscope.* By GUSTAVUS HARTRIDGE, F.R.C.S. Pp. xi., 128. London: J. & A. CHURCHILL. 1891.

We have read this Manual for Students with much pleasure. It is written in simple concise language, and describes most of the appearances seen by means of the ophthalmoscope in the healthy eye, and in diseased conditions of the organ. It contains many good diagrams illustrating the optical principles involved in the use of the instrument. The book is easily carried. Like the larger work on *Diseases and Refraction of the Eye*, by the same author, it can be well recommended for the use of practitioners or students of medicine.

